# Co‐Producing Training Resources to Improve Healthcare Experiences for Young Women and Reduce Gendered Health Inequalities

**DOI:** 10.1111/hex.70834

**Published:** 2026-08-20

**Authors:** Ana Alonso Curbelo, Emma Rigby, Alison Hadley, Sigi Joseph, Yvonne Kerr, Bethany Spain, Amy Simmons, Una Macfadyen, Sarah Dillon, Mar Sánchez Fernández, Laura Tinner

**Affiliations:** ^1^ University of Bristol Bristol UK; ^2^ Association for Young People's Health London UK; ^3^ University of Bedfordshire Luton UK; ^4^ RCGP Clinical Lead for Women's Health Scotland Edinburgh UK; ^5^ NHS Lothian Edinburgh UK; ^6^ Clackmannanshire Third Sector Interface (CTSI) Alloa UK; ^7^ Glasgow University Environmental Sustainability Team (GUEST) Glasgow UK; ^8^ NHS Edinburgh UK; ^9^ The Scottish Government Edinburgh UK; ^10^ The Young Women's Movement Edinburgh UK

**Keywords:** co‐production, experience‐based co‐design, gendered health inequalities, participatory methods, patient and public involvement, young women's health

## Abstract

**Background:**

Young women report feeling dismissed or unheard in healthcare settings. These experiences lead to mistrust, healthcare avoidance and ultimately contribute to gendered health inequalities.

**Objective:**

This project aimed to co‐produce with young women and key partners (e.g., healthcare professionals, experts in women's health and inequalities, third sector organisations and policymakers), resources to support healthcare professionals to recognise power imbalances and adapt practice to better meet young women's needs.

**Design and Contributors:**

Using an Experience‐Based Co‐Design (EBCD) framework, we conducted participatory creative workshops (one online and two in‐person) with three groups of young women (*n* = 24, aged 16–25). Workshops used arts‐based, accessible activities to explore both positive and negative healthcare interactions. Insights were then presented to 15 healthcare professionals in an online workshop, to co‐design practical resources.

**Results:**

The co‑design workshops led to the development of two linked resources: a co‑produced 2‑min animation and an accompanying training session for healthcare professionals. Young women identified three priorities for improving their healthcare experiences: (1) feeling heard and respected, (2) having agency and choice, and (3) continuity of care. Healthcare professionals emphasised the need for short, engaging and non‑judgemental resources. These insights informed the animation, which depicts themes drawn from young women's experiences. The training session, delivered and co‑created with young women who received facilitation training, incorporated structured engagement with the animation.

**Conclusion:**

This project demonstrates the value of co‐design in shaping meaningful, practical resources intended to support young women's healthcare experiences. The final product is now being shared with healthcare educators and services.

**Patient or Public Contribution:**

This project was co‐produced with young women and healthcare professionals from inception to dissemination via a Steering Committee and co‐production workshops. Interest holders from varying perspectives co‐designed the animation by shaping content and focus. Facilitation skills training was offered to all participating young women, with those who took part supported to remain involved through a young trainers' group. Healthcare professionals contributed insights that informed the resource's design, tone and messaging, ensuring it remained relevant and feasible within busy healthcare contexts. Steering Committee members were also invited to comment on manuscript drafts and support the development of this paper, including through co‐authorship.

## 1 Introduction

### 1.1 Gendered Health Inequalities and Young Women's Health

Gendered health inequalities remain a persistent and systemic issue in healthcare in the UK and internationally [1, 2]. A growing body of evidence highlights gaps in healthcare access, clinical research, diagnosis and treatment for women, often rooted in gender bias and the historical exclusion of women from medical research [3, 4]. These inequalities are compounded by intersecting systems of oppression such as racism, ageism, ableism and classism [5, 6, 7, 8, 9, 10, 11]. Qualitative research has shown that stereotypical assumptions contribute to these disparities, with healthcare professionals less likely to believe women's accounts of their symptoms and pain [12]. National data reflect these patterns. For example, the *Women's Health Strategy for England: Call for Evidence 2021–22* [13] received nearly 100,000 responses, with 84% of women reporting that they did not feel listened to by healthcare professionals. Similarly, the *Women's Health Survey (2022)* received more than 97,000 responses and highlighted widespread discomfort and mistrust [14].

These issues are particularly salient for young women during adolescence, a formative period for healthcare experiences and engagement [15, 16, 17], and therefore a pivotal stage for advancing gender equality across the life course [18]. Adolescence is commonly defined as the period between 10 and 19 years of age, although broader definitions extend into young adulthood (up to age 24–25) [19]. Experiences of adversity, social and economic disadvantage, and discrimination during this stage can have long‐lasting consequences for health in adulthood [20] [21]. Despite this, research focused specifically on young women's lived experiences remains limited. Our research with young women in Scotland [10, 22, 23, 24] set out to address this gap and consistently highlighted a pattern of what can be described as 'entrenched gendered ageism', a form of discrimination in which sexism and ageism operate simultaneously and inseparably. Young women across interviews and focus groups described feeling dismissed, patronised or not taken seriously, particularly in consultations relating to menstrual health, mental health and contraception [22, 24, 25].

These findings mirror broader national trends. *The Status of Young Women in Scotland 2022–23* report, drawing on survey responses from over 900 young women, found that only 30% rated their healthcare experiences positively, with many reporting that they were not believed, felt judged or faced barriers linked to both gender *and* age [26]. The *2024–25* report again identified healthcare as a site of perceived misogyny and ageism, with young women describing dismissive attitudes toward pain, limited understanding of reproductive and hormonal health and inadequate follow‐up or care [27].

These findings are also consistent with international evidence [28]. Qualitative reviews drawing on studies across multiple countries similarly highlight persistent gender biases in how women's symptoms, particularly pain, are interpreted and managed in clinical practice [29, 30].

Most healthcare professionals aim to deliver high‐quality, evidence‐based care, however they operate within systems historically shaped by patriarchal norms [31, 32]. Implicit bias—defined as subconscious attitudes that shape behaviour—can influence how symptoms are interpreted, how information is communicated and how seriously a patient's concerns are taken [29, 33]. While no comprehensive evidence base yet exists on treatment‑related inequalities for young women, this group's recurring experiences of dismissal have clear consequences for their trust in healthcare professionals, emotional wellbeing and long‑term engagement with healthcare services [22, 34, 35].

Emerging research has highlighted the need to actively address the intersecting barriers young women face [36, 37]. As the Young Women's Movement (a Scotland‑based organisation focused on gender equality and amplifying young women's voices) states: '*the health system needs to challenge medical misogyny and ageism, practise anti‐racism, dismantle ableism, and operate intersectionally*' [11]. This call to action underscores the importance of interventions that go beyond acknowledging gendered and age‑based health inequalities to meaningfully transform healthcare encounters and improve practice.

### 1.2 Policy and Practice Context

In 2021, Scotland became the first nation in the UK to publish a *Women's Health Plan*, marking a significant national commitment to addressing gendered health inequalities [38]. Now in its second phase [39], the Plan set out actions to raise awareness of women's health, improve access to healthcare across the life course and reduce inequalities in health outcomes for women and girls. These priorities closely align with policy developments elsewhere in the UK, including the *Women's Health Strategy for England*, which similarly aims to embed women's experiences and needs into healthcare design and delivery [13]. These national strategies align with wider international commitments to advancing gender equality in health, including the United Nations Sustainable Development Goal 5 and guidance from the World Health Organization (WHO) and the European Institute for Gender Equality (EIGE). Despite this policy momentum, a persistent gap remains between high‐level commitments and everyday clinical practice, particularly in primary care settings.

This project builds on qualitative research conducted to support the Women's Health Plan [10] which identified pervasive, intersecting age‐ and gender‐based discrimination in young women's healthcare experiences, particularly in primary care. Despite policy commitments to improving women's health, a gap persists between policy and practice, with limited training or frameworks to support healthcare professionals in addressing young women's needs. Existing youth‑focused frameworks, such as the Youth Friendly Services Framework [40], offer a useful foundation but do not sufficiently account for gendered and intersectional dimensions. Young women remain a relatively overlooked group within primary care despite frequent service use [41]. Therefore, what followed from our research was a participatory public involvement project to begin to address some of the issues highlighted by the young women in our qualitative research.

### 1.3 Project Aims

This public involvement project aimed to:

- Co‑produce training resources for healthcare professionals grounded in young women's lived experiences.
- Identify key priorities for improving healthcare encounters.
- Support healthcare professionals to recognise and address power imbalances.
- Contribute to policy and practice development on gendered health inequalities.

## 2 Materials and Methods

### 2.1 Project Design and Approach

This project adopted a participatory, collaborative design, underpinned by Patient and Public Involvement and Engagement (PPIE) principles [42, 43, 44], to develop training and awareness‐raising resources with young women and healthcare professionals.

**Figure 1:**
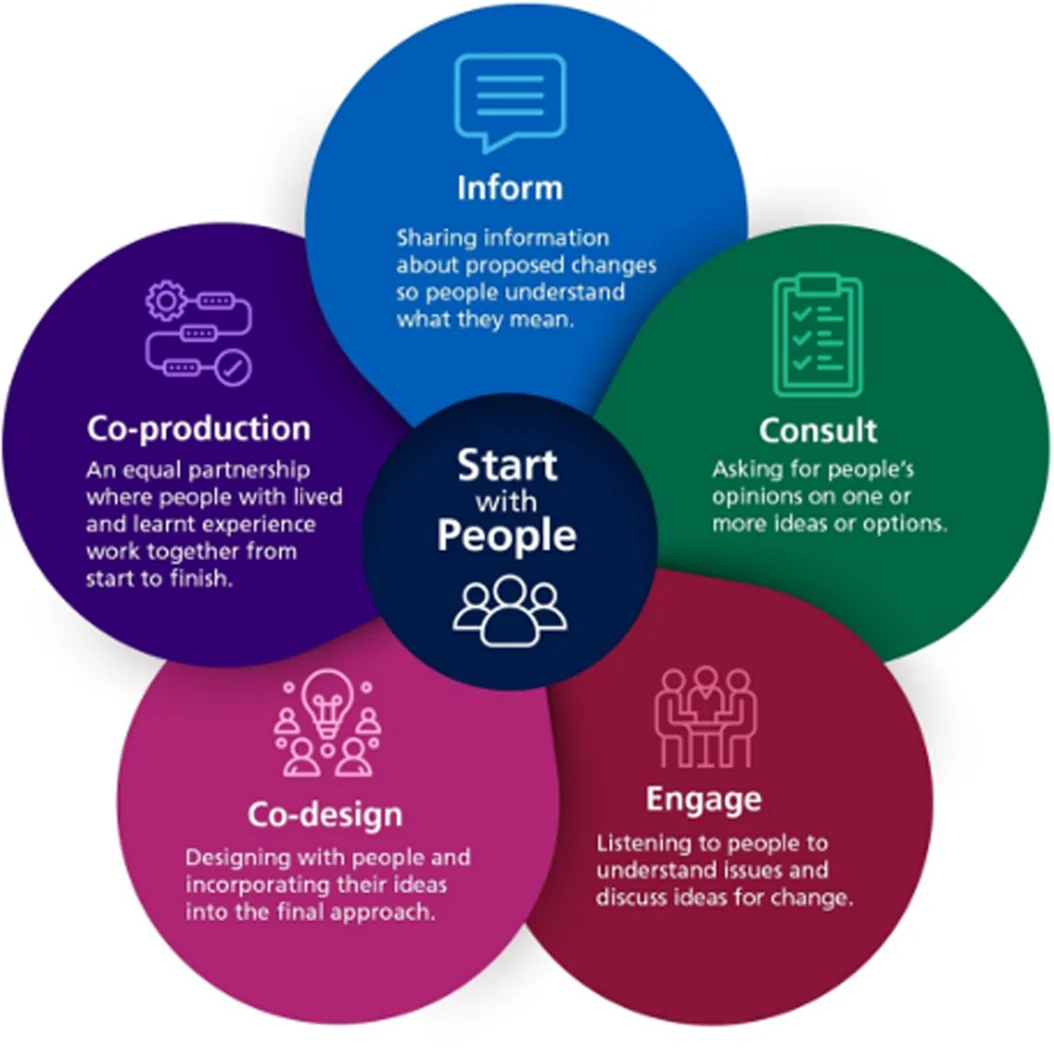
This diagram is from Annex A: Implementation of the statutory guidance for working in partnership with people and communities.

Participatory approaches, including co‐production and PPIE, are increasingly adopted as a way of centring lived experience in the design of healthcare interventions and training [44, 45]. Now mainstream within UK health research [46], these approaches position public contributors as equal partners and align with feminist ethics through an emphasis on power‐sharing, reflexivity and the creation of safe, supportive spaces for dialogue (see Figure 1) [47, 48].

There is growing evidence that interventions achieve better outcomes when communities are involved in their design and delivery [49, 50, 51]. Literature on youth participation further highlights that involving young people in research and policy is not only methodologically valuable, but constitutes a social justice outcome in its own right [52]. The co‐production process was guided by an Experience‐Based Co‐Design (EBCD) approach, which brings together service users and professionals to collaboratively improve services based on lived experiences [53, 54]. This involved iterative and flexible design, recruitment of diverse individuals across social and health contexts and the inclusion of multiple collaborators to broaden perspectives [55].

Given that General Practice (i.e., primary care practitioners, including GPs, nurses, advanced practitioners, pharmacists, clinical support staff and community‐based healthcare workers who deliver first‐contact care) is the most frequently accessed healthcare setting for young women, the resource was co‐developed with young women and healthcare professionals working in that setting. Although, the training resources will hold relevance to healthcare professionals in other settings.

**Figure 2:**
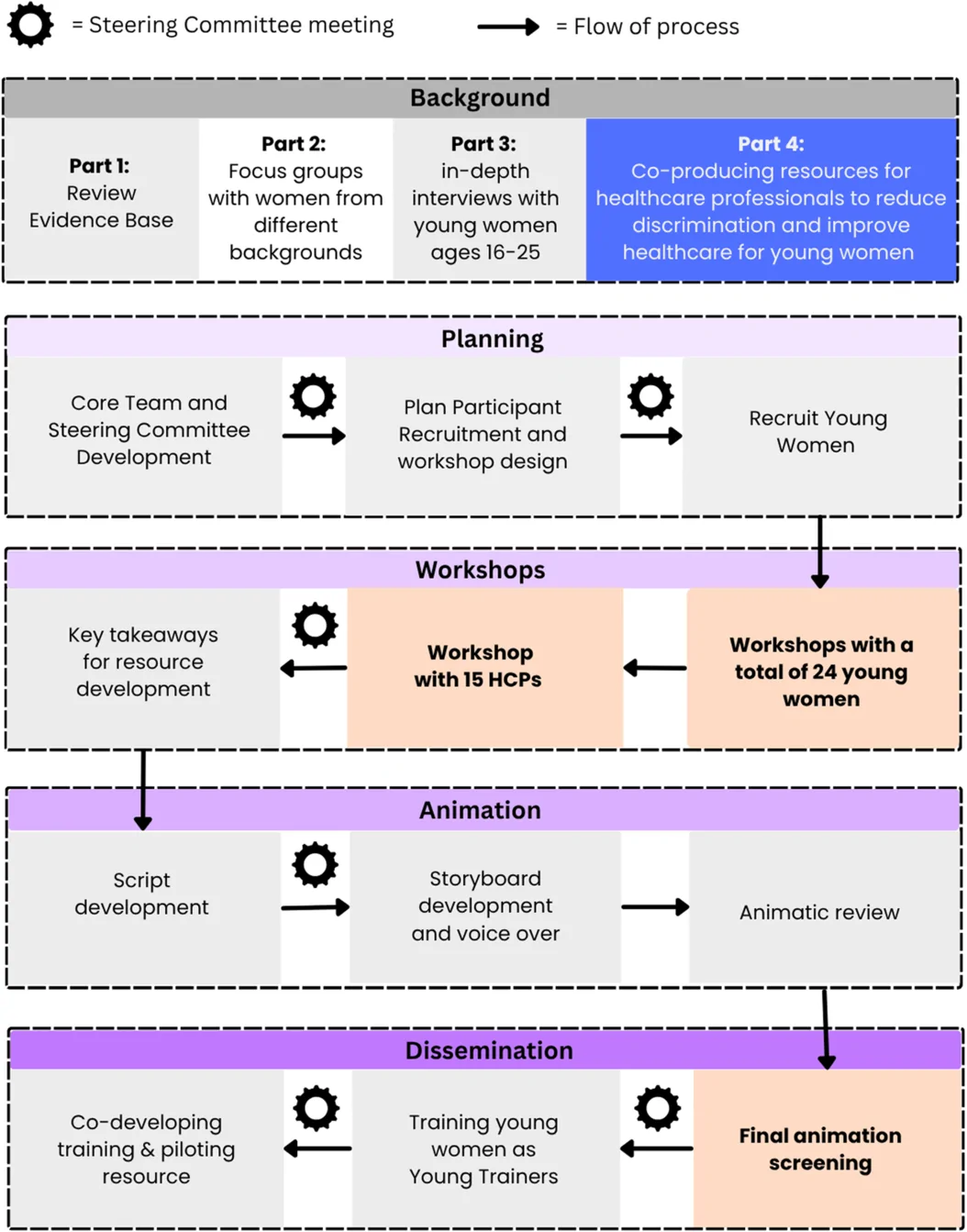
Overview of the co‑production process.

We provide an extensive reflexive account of working with young people and healthcare professionals, including the power dynamics involved in these projects, in a separate paper [56]. Figure 2 below presents a flowchart summarising the co‑production process, from initial planning and recruitment through to workshop delivery, resource development and pilot testing the training.

#### 2.1.1 Ethics and Safeguarding

As confirmed by the lead institution's ethical approval co‐ordinators, we did not require formal ethical approval for this project given it is not research but instead a public involvement and engagement project. Nonetheless, ethical principles remained central to the project and we abided by the ethical frameworks of our institutions and funding bodies. Verbal informed consent was obtained from everyone involved, ensuring they fully understood the purpose of the co‐production process, what participation involved and their right to withdraw at any time without consequence. Particular attention was paid to safeguarding, with protocols in place to support young people who might experience distress during discussions of sensitive topics. Our collaborator who is a UK leader in engagement with young people acted as the safeguarding lead.

### 2.2 Project Steering Committee

From the outset, the project was co‐developed with partners from policy, practice and lived experience, who contributed to the funding application and shaped the research aims. We designed the project to be underpinned by a Steering Committee, which was inspired by similar co‐production projects [53, 57, 58, 59, 60, 61, 62, 63]. Members were recruited through targeted calls via established networks, with selection guided to ensure representation across lived experience, healthcare professional backgrounds and policy and third sector perspectives.

Rather than serving solely as an advisory body, the Steering Committee was integral to the participatory approach of the project. Established in January 2025, the Committee brought together a range of perspectives (*n* = 9), including three young women with lived experience, representatives from General Practice, Sexual Health, Paediatrics, Gynaecology, Women's Health Policy, Community and Voluntary Sector advocacy and public health researchers. All members received a Steering Committee Terms of Reference (see Supporting Information: Material S6), which clarified roles, expectations and boundaries. All members of the Steering Committee were women, although this was not an intentional selection criterion. Five online meetings were scheduled over the course of the 9‐month project, each focusing on different topics to maximise input (see Table 1).

**Table 1:** Summary of Steering Committee schedule.

Month	Stage	Focus/Content	Key feedback and impact
March	Planning and recruitment	Introductions and project overview Terms of Reference agreed Discussion of recruitment strategies for young women and healthcare professionals. Project roadmap reviewed	Target recruitment through community groups to reach marginalised young women; Highlighted accessibility needs; healthcare professionals' recruitment to include support staff and young trainees; Inclusive language finalised for recruitment flyer; Project name and logo chosen (“SHIFT”)
April	Workshop planning	Recap of project Discussion of planned workshop content and activities (young women & healthcare professionals) Workshop recruitment updates	Young women's workshops to use interactive creative methods; Expand healthcare professionals recruitment beyond GPs and nurses; Use a short survey to broaden healthcare professionals reach.
July	Post‐workshop animation development	Presentation of animation brief, draft script, and storyboard Review of style samples Feedback on tone, content and audience	Script revised; Audience clarified via title page; Tone refined to balance empathy, humour and non‑blaming approach
August	Dissemination and training	Showed the animatic (moving story board) and refined language and made final tweaks. Update on facilitation training of young women Planning pilot training session	Identified groups of healthcare professionals' for pilot; Agreed training should be online; Shared contacts for recruitment; Agreed key training principles and scenario topics (e.g., mental health, menstrual health)
October	Animation Screening	Screened the final animation Discussed the pilot training session, including approach, content and delivery logistics	Confirmed next steps for pilot rollout; Agreed on evaluation plans and how feedback would be gathered; Reviewed dissemination opportunities and next steps for circulating the animation strategically

During meetings, we discouraged the use of formal professional titles to flatten traditional hierarchies and create a more welcoming space. Relationship building was actively nurtured through informal communication alongside facilitation strategies designed to create a supportive, collaborative environment during meetings. We also shared pre‐meeting and post‐meeting notes using visually engaging newsletters to update the team, setting the stage for discussion and ensuring members were informed about decisions made following meetings (see Supporting Information: Material S4). We included short surveys in the pre‐meeting newsletters (see Supporting Information: Material S4). Although originally designed to enable contributions from members who might be unable to attend, in practice it also served as a space where the Steering Committee could signpost resources and provide suggestions that we did not have time to discuss in the meeting. Survey engagement was consistently high which meant that responses could be used to prepare more focused agendas and anticipate areas requiring deeper discussion. By combining these strategies, the Steering Committee became an active, collaborative force, directly influencing project decisions.

### 2.3 Co‐Production Workshops

Three co‐production workshops were conducted with young women aged 16–25 (two in‐person, one online, *N* = 24) and one workshop was subsequently held with healthcare professionals online (*N* = 15).

#### 2.3.1 Young Women's Workshops

Young women were recruited to the online workshop through an online registration survey distributed through the networks of the project team and Steering Committee. Contributors provided limited demographic details (gender, age, postcode) and contact information to allow targeted selection and to prevent ineligible ‘imposter’ participation, that is, individuals who are not young women living in Scotland. Having a formal registration process (particularly providing postcodes which can be checked if they are residential) has been found to partially address this issue [64] and we do not believe we involved any ineligible contributors.

For in‐person workshops, recruitment was targeted through community partners to reach groups facing intersectional barriers, focusing on ethnicity, LGBTQ+ identities, disability and rurality. Working with established community organisations during recruitment was key to build trust and reach young women potentially experiencing marginalisation and who may be less likely to engage through open recruitment methods. This approach was intended to support engagement with groups experiencing intersecting forms of disadvantage. Opportunities to participate were advertised through multiple channels, including word of mouth, email and flyers. Expressions of interest could be submitted via an online form, email, call or text.

Eligible contributors received detailed information and a chance for an initial online discussion before confirming involvement and were compensated for their time at NIHR Public Involvement rates [65]. Accessibility considerations included language, cultural sensitivity and support for childcare and travel expenses. Information materials were designed to be clear and accessible and provision was also made within the project budget for translation or interpretation support if required; however, no contributors requested these services. We aimed to be as inclusive as possible while balancing the focus of the project on young women and therefore we included the following text on the flyer, as approved by the Steering Committee: ‘*This project is inclusive of Trans and Intersex women, as well as non‐binary and gender fluid people who are comfortable in a space that centres the experience of women.’*

**Figure 3:**
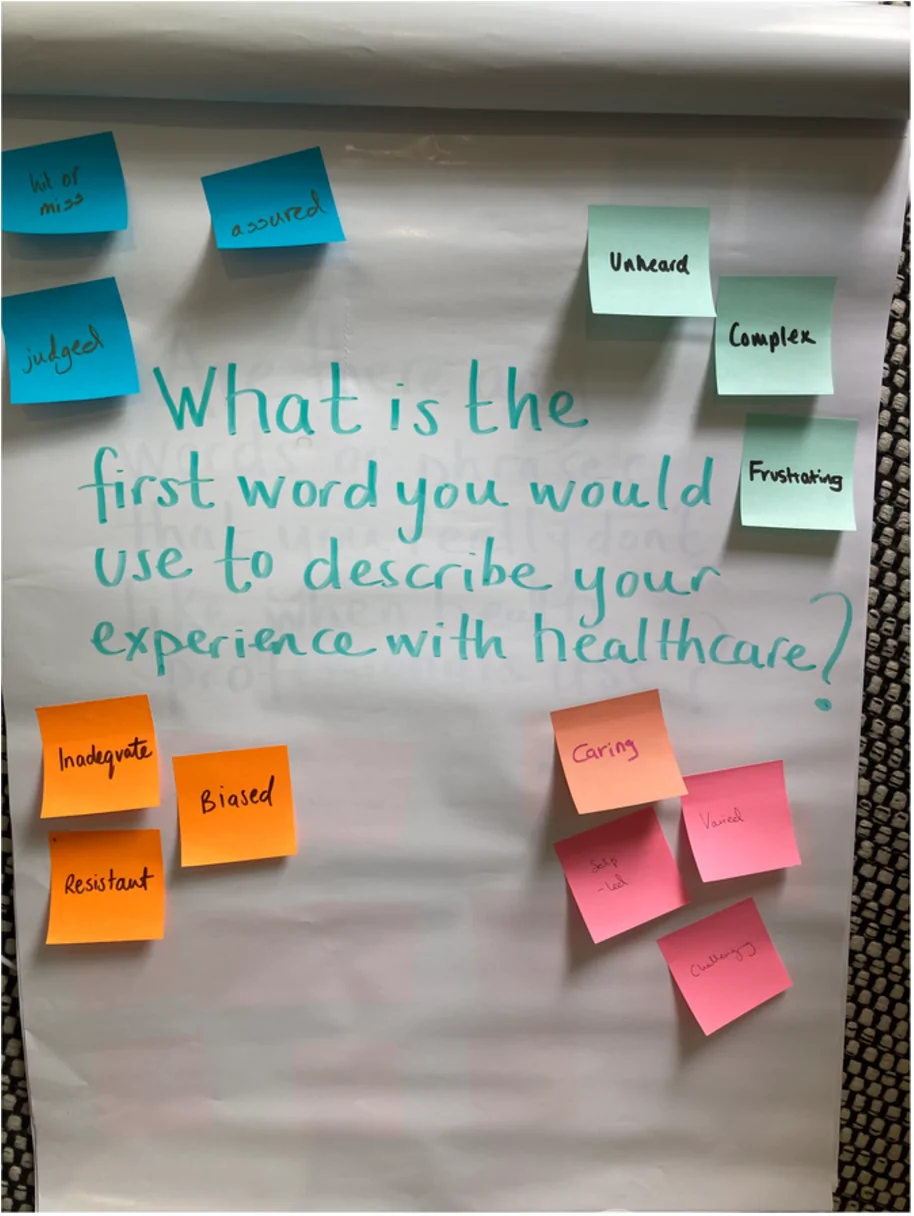
Creative activities used within young women's workshops.

**Figure 4:**
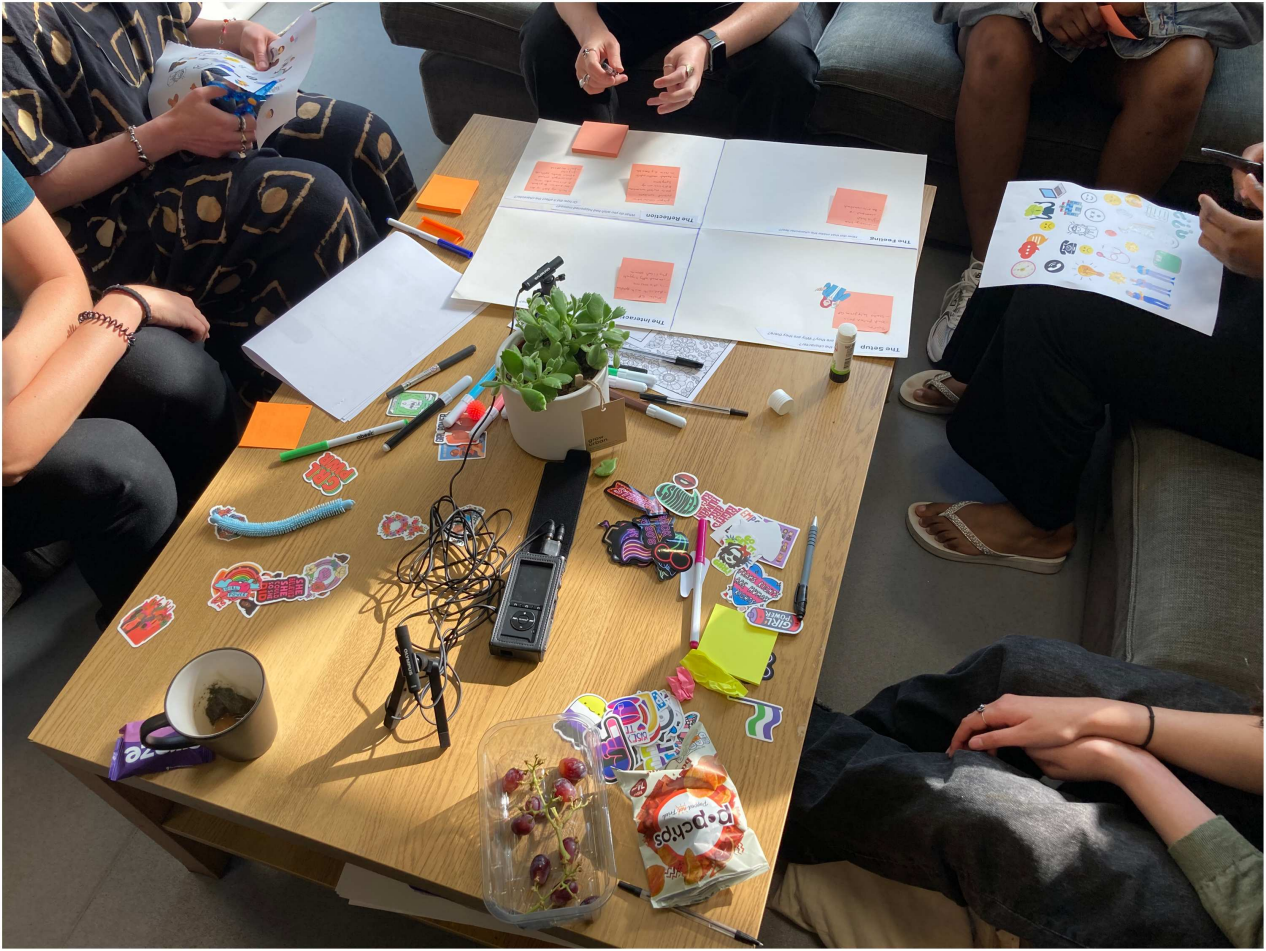
Creative activities used within young women's workshops.

The young women's workshops explored healthcare experiences, identified key challenges and generated ideas for improvement to inform the content of the resources. These workshops were designed and facilitated by the project team in collaboration with our community partners [66]. Intentional decisions were made to create a welcoming and supportive environment: in‐person sessions were hosted in community venues familiar to our contributors; the layout of rooms encouraged collaboration and minimised hierarchy; refreshments and fidget toys were provided and spaces for breaks were available (Figures 3 and 4) [66]. Multimodal approaches supported young people to share perspectives visually and verbally, as well as enabling quieter individuals to contribute fully. Online workshops mirrored these principles through using breakout rooms, interactive polls and the chat function to ensure that everyone had different available ways to engage.

All young women who attended the workshops were given the opportunity to express an interest in participating in a facilitation training programme and supporting the development of a prototype training workshop for healthcare professionals based on the issues raised during the co‐production workshops. The facilitation training programme covered communication and facilitation skills and was delivered across three sessions. Six young women attended the first two sessions, with four completing all three. Following the training, two young women worked with the project team to co‐design and co‐deliver the pilot training workshop for GPs, helping to shape its content and delivery. Everyone completed feedback forms to support our reflection.

#### 2.3.2 Healthcare Professionals

Healthcare professionals were recruited through professional networks using an online registration survey. Recruitment was supported through Royal College mailing lists, professional newsletters distributed across Scottish health boards and networks of project partners and Steering Committee members. Practical considerations included scheduling meetings with at least 6 weeks' notice, offering funding to support clinical staff backfill where possible and encouraging participation from diverse perspectives, including asking practice managers to encourage senior and male colleagues to attend.

The focus of the online workshop with healthcare professionals was to share findings from young women's workshops, facilitate a discussion about barriers and opportunities for delivering inclusive care to young women and gather feedback on the tone, style, language and format of the planned resources. The project team facilitated discussions on how the resources could best reflect young women's experiences while remaining relevant and practical for clinical settings. To support these conversations, our media partner presented early concepts, including potential formats, storylines and visual approaches, which were used as prompts for discussion and feedback. The workshop was led by the project team with support from our GP partner. Mirroring the young women's workshops, facilitation focused on creating a non‐hierarchical and reflective space where healthcare professionals could share their experiences without fear of judgement. We used interactive methods such as “chat waterfalls” (where contributors post responses simultaneously) and scenario‐based discussions to encourage participation and generate ideas for refining the resources.

A detailed overview of workshop activities, discussion topics and facilitation methods is provided in Table 2.

**Table 2:** Overview of workshops.

Young women's workshop	Healthcare professionals' workshop
1. **Introductions and group agreement**: icebreaker activity, setting ground rules around confidentiality, consent and self‐care, clarifying workshop aims and the resource to be developed. 2. **Exploring first impressions of healthcare:** whole group activity using flashcards and Post‐Its to understand healthcare experiences. 3. **Storyboarding healthcare experiences:** small group exercise creating characters and narratives to illustrate how young women may feel in healthcare consultations, followed by group sharing. 4. **“Flip the Script” activity**: groups discussed real‐life scenarios of dismissive responses from healthcare professionals and co‐created affirming, person‐centred alternatives. 5. **Ideal consultations and advice to peers**: whole group discussion on what an ideal healthcare interaction should look like and what advice participants would give to a friend. 6. **Closing reflection**: participants rated the session, identified next steps, and were thanked for their contribution. Facilitators debriefed following the session.	1. **Introductions and project overview:** icebreaker activity and overview of project aims, situated within the Women's Health Plan and recent research on young women's healthcare experiences. Facilitators emphasised that challenges faced by healthcare professionals were recognised and valued. 2. **Young women's perspectives:** presentation of themes and quotes from the young women's workshops (feeling heard, choice and clarity, ongoing care), followed by group discussion of how these issues might be addressed in practice. 3. **Designing a resource:** group discussion of what kind of training resource would be feasible and engaging for healthcare professionals, with input from Media Co‐op on potential formats (e.g. short animation, takeaway messages). 4. **Closing reflections:** participants shared a word or phrase about their experience, gave feedback via polls, and were thanked for their contributions

## 3 Key Messages for the Training Resources

Workshop outputs were reviewed collaboratively by the project team and Steering Committee to identify recurring priorities and key messages. Through this iterative process, areas of consensus and key themes were refined and used to guide the development of the animation and training resources.

### 3.1 Key Themes from Young Women's Workshops

A total of 24 young women took part in the workshops. Contributors ranged in age from 16 to 25 years and came from both urban and rural areas with differing levels of socioeconomic deprivation. Although detailed demographic information was not collected (including disability, health conditions and ethnicity), recruitment through community organisations aimed to engage young women who may experience marginalisation or intersecting barriers to participation. This was reflected in workshop discussions, which highlighted experiences of racism, ableism and other forms of discrimination in healthcare.

The co‐design workshops with young women, conducted both online and in‐person, revealed three central themes about their healthcare experiences that they wanted to be integrated into the training resources:

- Feeling heard and respected: Being listened to and believed was central to positive experiences. While young women recognised the time and resource pressures faced by healthcare professionals, dismissal or minimisation undermined trust, while respectful engagement, regardless of immediate outcomes, made young women feel supported.
- Agency and choice: Young women valued clear information, shared decision‐making and being offered options. They emphasised care that considered their wider lives, beyond reproductive health, and support to self‐advocate, particularly when accessing healthcare independently.
- Continuity of care and ongoing care: young women expressed a desire for continued support beyond single appointments, particularly for issues that required monitoring or follow‐up. This often required explicit invitation to return to primary care later if the health issue had not gone.

### 3.2 Key Themes From the Healthcare Professional's Workshop

A total of 15 healthcare professionals took part in the workshops, including 13 GPs and two Nurse Practitioners. Contributors worked in practices across Scotland and represented a range of geographical and socioeconomic contexts. One male healthcare professional participated, with the remainder identifying as women. Healthcare professionals were presented with the themes identified by young women. Many expressed surprise at the extent to which young women described feeling dismissed or unheard, highlighting a gap between healthcare professionals' perceptions and young women's lived experiences. For some, this prompted reflection on how routine clinical practices, time pressures and implicit biases might unintentionally contribute to these experiences.

This reflection informed consensus on the resource design. Healthcare professionals agreed that it should be:

- Concise and focused, reflecting the time constraints of clinical practice.
- Accessible, using clear, inclusive language and visuals suitable for diverse audiences.
- Approachable, adopting a light‐hearted tone where appropriate to encourage engagement.
- Non‐judgemental, recognising systemic pressures and offering constructive, practical strategies rather than critiquing individual practice.

### 3.3 Co‐Produced Training Resources

Two linked resources were co‐produced: (1) a short, narrative animation portraying young women's healthcare experiences, and (2) an accompanying training session co‑delivered by young women who received facilitation skills training as part of the project (See Supplementary Material S1).

#### 3.3.1 Animation

**Figure 5:**
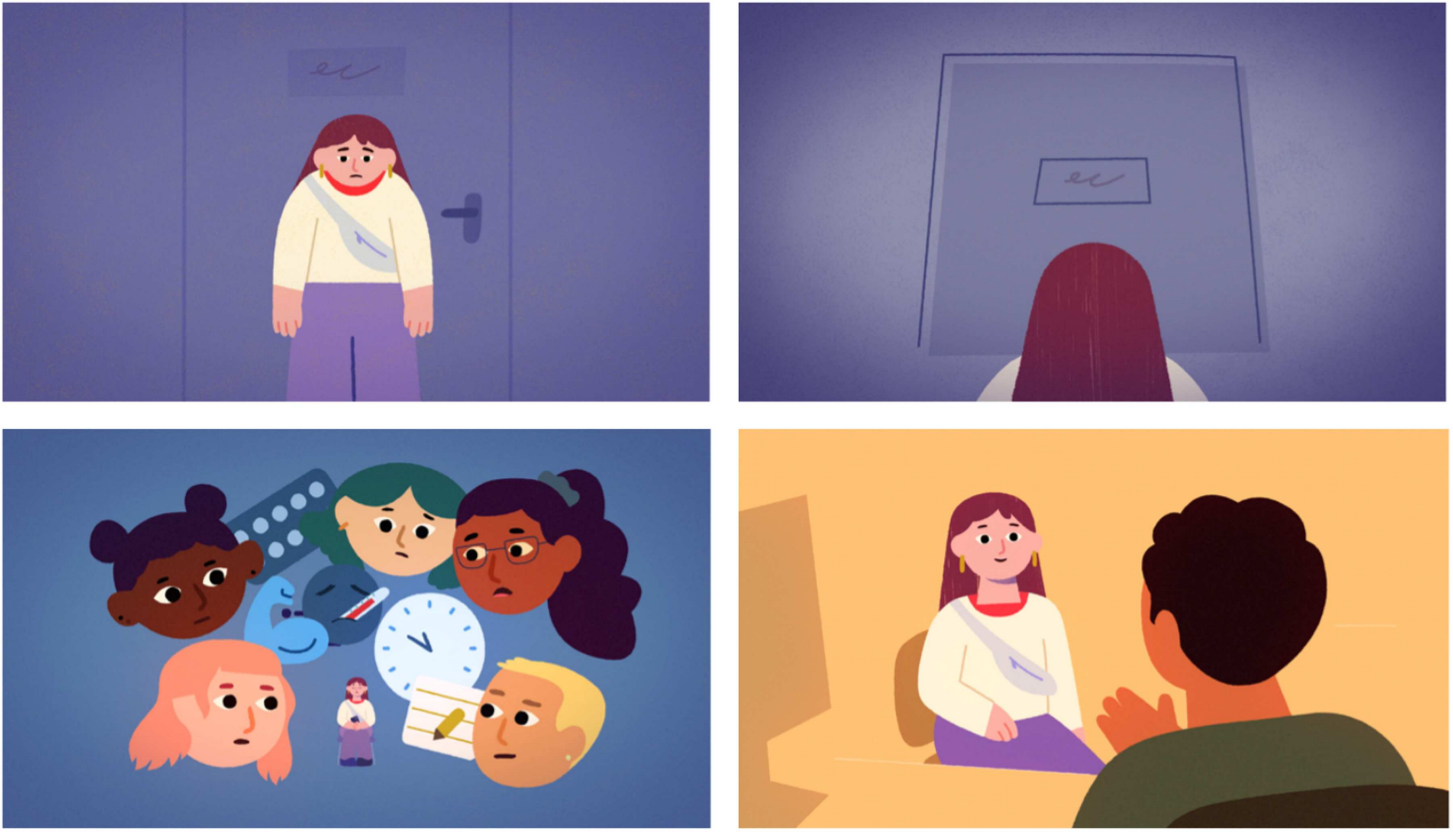
Frames from the co‑produced animation. Selected frames from the 2‑min animation illustrating the journey of “Erin,” a fictional young woman navigating a primary care consultation. Including the key messages for healthcare professionals.

**Figure 6:**
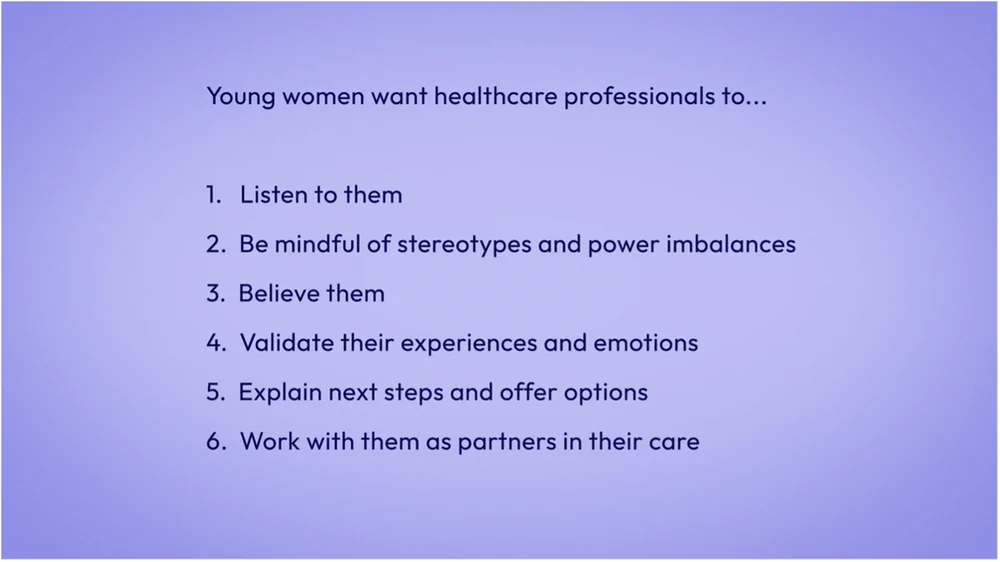
Frames from the co‑produced animation. Selected frames from the 2‑min animation illustrating the journey of “Erin,” a fictional young woman navigating a primary care consultation. Including the key messages for healthcare professionals.

Based on feedback from healthcare professionals, the Steering Committee and young women, a short narrative animation was created by our media partner, which was an organisation specialising in accessible, engaging media for health, education and policy. The animation follows Erin, a young woman, as she prepares to see her doctor—using the language used by young women in the workshops, who commonly used this term to refer to their GP or primary care clinician—while reflecting on past negative experiences and advice from friends on how to best use the appointment. During the consultation, Erin experiences a positive interaction, demonstrating empathy, collaboration and validation from the healthcare professional. The story models simple, practical behaviours that foster inclusive care without adding to doctors' workload. The tone remains light‐hearted, focusing on potential solutions rather than critiquing current practice, concluding with key takeaways and links to further resources (see Figures 5 and 6).

Throughout development, the project team and Steering Committee provided iterative feedback on the script, storyboard, visual style and key messages, ensuring the animation reflected young women's experiences and avoided costly post‐production changes. To extend the co‐production approach, the main character is voiced by a young woman who participated in one of the workshops, recruited through our invitation to all contributors to audition. The successful contributor was paid at a professional actor rate and provided with a reference by the media company to include on her résumé.

#### 3.3.2 Training Session

To expand the use of the animation, we co‐developed an accompanying training session. The training was co‐developed and co‐delivered with two of the young women who had been trained as young facilitators working closely with the project team. The content was overseen by the Steering Committee. The training had the following learning objectives:

- To deepen understanding of young women's experiences in healthcare by sharing key insights from research and young women's lived experience.
- To explore opportunities and barriers for shaping quality healthcare experiences for young women.
- To provide access to tools and resource(s) that can support in delivering the best possible healthcare experiences for young women.

The training session was 90 min long and included a screening of the animation, interactive activities and case studies that encouraged reflection on areas where young women frequently reported misunderstanding or dismissal, such as contraception, pain and mental health. We piloted the training session online. Steering Committee members supported recruitment, targeting primary care trainers and trainees across selected regions in Scotland, with funding offered to cover clinic time where necessary.

The pilot allowed us to gather feedback on the potential integration of the session into training for primary care staff. A total of 14 GPs and GP trainees from Scotland attended the session with all saying that it was either relevant or extremely relevant to their practice and that they could take the learning from the session into a healthcare setting. One healthcare professional on the Steering Committee also noted that the process *'contributed to my own learning and understanding of issues not commonly discussed in healthcare management or delivery.'* These early indicators of feasibility and relevance suggest that the resources could be successfully integrated into wider professional development initiatives across primary care.

## 4 Discussion

### 4.1 Reflections on the Co‐Design Process

Feedback from Steering Committee members underscored the value of the co‑design approach, both for the project and for contributors themselves. Members described the process as '*engaging', 'fun*' and '*effective*'. Others highlighted the benefits of hearing diverse perspectives, '*sharing ideas, learning new concepts*' and emphasised the supportive environment: '*everyone felt listened to and their opinions valued*.' These reflections, gathered through a survey after the project concluded, illustrate the strengths of participatory, co‑production approaches [67] in developing resources to improve young women's healthcare experiences. Phase 2 of the Women's Health Plan continues to stress the importance of inclusive, person‑centred approaches and the active involvement of people with lived experience in shaping services [39]. Iterative feedback loops were central to the co‐design process. Only through sustained, reflexive collaboration was it possible to co‐produce resources that were both acceptable to healthcare professionals and authentic to young women [42, 63]. Lived experience was woven into the development process, and Steering Committee members were invited to develop and co‑author this paper [68, 69]. Most of them embraced the opportunity and provided comments on the manuscript drafts.

We also ensured we provided additional opportunities for the young women, that were not purely about the development of the training resources and meant that they were either gaining skills or experience and they were paid for those activities. These opportunities included the facilitation training which was broad and can be used in a range of industries, voice acting in the animation, presenting at dissemination events and blog writing [70]. Given the group we were working with (young women), we saw these offerings as beneficial in building our trust and relationship with the contributors, increasing their confidence and skills and reducing the risk of tokenism or extractive public involvement [71]. We remain in touch with the young women and offer further opportunities for involvement in other projects or events as they arise.

Although the broader impact of the animation on clinical practice has yet to be evaluated and remains an important next step, early feedback from the prototype training session is encouraging. We believe the co‐production process was fundamental to the content being relevant and well received [42]. The animation has been disseminated through partner organisations, policy and professional networks and channels, generating expressions of interest in the accompanying training from multiple organisations. It was also featured in an episode of the Primary Care Knowledge Boost podcast [72], which discussed the project findings and practical implications for healthcare professionals. It has also been incorporated into Phase Two of the Women's Health Plan (2026–2029) and was selected for screening at the MOVE Summit in Edinburgh. We see these outputs as a catalyst for further work to improve practice and promote health justice for young women.

### 4.2 Limitations

Co‑production is resource‑intensive and requires sustained coordination [73]. Working with multiple partners required continuous reflexive practice to attend to power dynamics and positionality.^1^ This was supported through regular project team debriefs, iterative feedback from contributors and Steering Committee members and ongoing consideration of power dynamics throughout the co‐production process.

Despite intentional strategies to flatten hierarchies (e.g., discouraging formal titles, supportive ground rules), power dynamics (including age‐based differences) cannot be fully removed [74, 75]. One Steering Committee member also noted that greater clarity about members’ backgrounds might have supported more directed discussion, underscoring the balance between inclusivity and the practical need for structure. While accessibility and flexibility were prioritised throughout, differences in perspectives, particularly between healthcare professionals and young women, also sometimes required careful negotiation.

A further limitation arises from the scope of the resource we co‐produced. Both young women and healthcare professionals identified structural constraints, such as limited time, challenges with continuity and wider organisational pressures, that extend beyond what training resources alone can address. As intermediaries, we prioritised aspects we could meaningfully influence. While pragmatic, this risks sidelining structural concerns likely to have the greatest impact on health justice [76], and reflects the limitations of small‐scale individual level interventions, which form only one component of a wider multilevel approach to reducing inequalities [77]. Although a range of lived experiences informed the animation, we did not collect a range of demographic data and recognise this as a limitation. Some groups, such as those with limited digital access or language support, may be under‐represented.

### 4.3 Future Directions

The animation and accompanying training materials show strong potential for integration into continuing professional development (CPD), medical and nursing education and practice‐level training. While the prototype training session with GPs demonstrated promise, future scaling may require adaptation for different healthcare settings. The flexibility of the prototype training session provides a practical means to tailor the resource to specific contexts or populations. There is also scope to extend the resource beyond clinical training. Adaptations for schools, colleges and youth settings could support young people's healthcare autonomy from early adolescence. Similarly, the resource could be used with parents and carers to build understanding of the support young women may need when navigating healthcare and transitioning towards adult health autonomy.

Next steps include dissemination of the animation and seeking opportunities to deliver the training session alongside possible integration into CPD, undergraduate and postgraduate curricula and wider policy initiatives. While early feedback from the pilot workshop and Steering Committee is promising, the impact of the resource on healthcare practice and young women's experiences has yet to be established. Research is essential to understand its effectiveness, and to support longer‑term efforts to reduce inequalities and promote more equitable, person‑centred care.

## 5 Conclusion

This project co‐produced training resources with young women and healthcare professionals with the aim of improving young women's healthcare experiences. A structured participatory model underpinned the work: a diverse Steering Committee guided decision‑making; co‑design workshops shaped content, tone and messaging; and young women were offered facilitation‑skills training, enabling those who participated to take on active roles in delivery and continue developing transferable skills. Through collaborative workshops and engagement with a diverse Steering Committee, the project embedded young women's and healthcare professionals' perspectives into both the content, design and delivery of the resources. This project therefore contributes to the growing evidence on co‑design and PPIE in healthcare, demonstrating how participatory approaches can generate training materials that are contextually relevant, inclusive and feasible to implement.

## Author Contributions

**Ana Alonso Curbelo:** conceptualisation, investigation, funding acquisition, methodology, visualisation, writing – review and editing, project administration, writing – original draft. **Emma Rigby:** conceptualisation, investigation, funding acquisition, writing – review and editing, visualisation, project administration, resources, validation. **Alison Hadley:** investigation, writing – review and editing, supervision, validation. **Sigi Joseph:** investigation, writing – review and editing, supervision, validation. **Yvonne Kerr:** investigation, writing – review and editing, supervision, validation. **Bethany Spain:** investigation, writing – review and editing, supervision, validation. **Amy Simmons:** investigation, validation, writing – review and editing, supervision. **Una Macfadyen:** investigation, writing – review and editing, validation, supervision. **Sarah Dillon:** investigation, funding acquisition, writing – review and editing, validation, supervision, methodology. **Mar Sánchez Fernández:** investigation, writing – review and editing, supervision. **Laura Tinner:** conceptualisation, investigation, funding acquisition, writing – review and editing, validation, methodology, formal analysis, project administration, supervision, resources.

## Ethics Statement

This is a public involvement project and therefore did not require formal ethical approval. The research that preceded this work was approved by the Scottish Government ethical procedure.

## Conflicts of Interest

The authors declare no conflicts of interest.

## Supporting information


Supporting File 1

Supporting File 2

Supporting File 3

Supporting File 4

Supporting File 5

Supporting File 6

Supporting File 7

## Data Availability

Research data are not shared. This was not a research study and therefore data are not available for further use by researchers.

## Footnotes

1 We provide an extensive reflexive account of working with young people and healthcare professionals, including the power dynamics involved in this project, in a separate paper [56].
