# Supplementary material for: Co‐Producing Training Resources to Improve Healthcare Experiences for Young Women and Reduce Gendered Health Inequalities
Source: Health Expect. 2026 Aug 20;29(4):e70834. doi: 10.1111/hex.70834 (PMC13492181; doi:10.1111/hex.70834)
Supplement: Supplementary file 1 [file HEX-29-e70834-s001.pdf]

SHIFT workshop Feedback Form

We'd like to know your thoughts on the workshop so we can continue to improve.

How would you rate your overall experience at the workshop?

Very Bad ○ ○ ○ ○ ○ **Excellent**

Did you feel that you have the chance to contribute as much as you wanted?

Yes No

What was your favourite part of the workshop? (e.g., activity or topic)

Is there anything that could have made the workshop better?

Thank you for your participation and continued support!

SHIFT

Improving healthcare experiences for young women
