# Supplementary material for: Co‐Producing Training Resources to Improve Healthcare Experiences for Young Women and Reduce Gendered Health Inequalities
Source: Health Expect. 2026 Aug 20;29(4):e70834. doi: 10.1111/hex.70834 (PMC13492181; doi:10.1111/hex.70834)
Supplement: Supplementary file 2 [file HEX-29-e70834-s002.pdf]

SHIFT workshop 1 - online 15/05/2025 4.30pm-6.30pm (2 hours)

Objective 1: To understand the key issues driving healthcare experiences for young women

Objective 2: To agree on the key messages that should be included in a training resource for healthcare professionals

Activity	Details	Time and Facilitator	Tech
**Icebreaker**	• Banana Canva for arrival<br>• Feelings check in<br><br>*Icebreaker*<br>- What's your first name and what did you have for lunch?	**Facilitators introduce themselves.**<br>4.30-4.40 (10 mins)	Slides 1-2 in Canva presenter mode<br><br>Banana choice in the chat<br><br>Launch feelings check in poll in Zoom<br><br>Put question in chat
**Set up**	• Why are we here? What are we hoping to achieve?<br>• What activities will we be doing?<br>• What will the workshops be used for? The resource we hope to create<br>• Looking after ourselves<br>• Consent and confidentiality – you have read the information sheet and by being here consent to taking part and us recording and using these discussions to help make our resource. All names and places will be removed so nobody except us will know what everyone has said. If you want to withdraw at any point you can, but we can't remove all the things you've said, but if there is something specific you'd like us to remove we can do that.	**Facilitator 1**<br>4.40-4.50<br>(10 mins)	Slides 3-10 in Canva presenter mode
**Activity 1**<br><br>4.50 - 5.00 (10 minutes)	**START RECORDING**<br>**Whole group work**<br>*Using chat*	**Facilitator 2**	**Start recording, but others remind each other**<br><br>Either using Canva presenter view or put

	Question 1, 30 seconds to write and then click enter at the same time:<br>• What is the first word you would use to describe your experience with healthcare?<br>**Facilitators briefly reflect**<br><br>*Question 2: 30 seconds to write and then click enter at the same time::*<br>• Are there any words or phrases that you really don't like when healthcare professionals use?<br><br>**Facilitators briefly reflect**		questions in the chat.<br><br>**Chat waterfall** in zoom. Give people a specific amount of time to answer, 30 seconds, and ask people to type up a response. Then say GO and everyone presses enter at the same time. This avoids sharing the screen, and also its a bit stressful when people can read as you're typing.
**Activity 2**<br>5.00 - 5.25<br>*25 minutes*	**Small breakout rooms with facilitator in each**<br>*Storyboarding young women's healthcare experiences – 20 minutes*<br><br>• Each group has a 3-panel storyboard with the title 'When young women don't feel heard in healthcare settings'<br>• Prompts and questions, with images they can add into the panels to create a story that shows how a young woman might experience going to see a doctor or nurse in primary care.<br><br>*Come back as a group and share the story in one minute (the facilitator can do this on the group's behalf, or if someone wants to)*	**Facilitator 2 introduces session and shares example story board**<br><br>**Facilitators 2 and 3 facilitate a group each and Facilitator 1 move between.**<br><br>**Media partner will be observing in one**<br><br>**20 mins in rooms**<br><br>**5 minutes sharing**	Presenter mode in Canva, Facilitator 2 sharing screen:<br>• *Blank storyboard – slide 11*<br>• *Example filled in storyboard – slide 12*<br><br>**Facilitator 1 starts and ends breakout rooms –**<br><br>Interactive mode (facilitator sharing not full screen):<br><br>Group 1 storyboard – slide 13<br><br>Group 2 storyboard – slide 14<br><br>**Restart recording for group discussion**
**Break – 5 minutes**			
**Activity 3**	**Small break-out groups**	**Facilitator 3 introduces and shows example**	Facilitator 3 Canva presenter mode to show example.

5.40 - 5.55<br><br>**(25 minutes)**	*Flip the Script – 15 minutes*<br>• Look at each SITUATION card created using quotes from our findings (e.g. “A 20-year-old woman goes to the doctor complaining of persistent fatigue and body pain.”).<br>• Match it with the RED card that reflects a real healthcare response.<br>• As a group, write a GREEN card on a green Post-it note — this is your version of a helpful, affirming, and person-centred response from a healthcare professional.<br><br>*Once all scenarios have GREEN responses, come together as a full group to compare and discuss. - 10 minutes*	**Facilitator 3 and Facilitator 2 facilitating each group**<br><br>15 mins in breakout rooms<br><br>10 mins discussion	**Facilitator 1 puts us into same breakout groups.**<br><br>Interactive mode (facilitator sharing not full screen):<br><br>Group 1 – Slide 15<br><br>Group 2 – Slide 16<br><br>**Restart recording for group discussion**
**Activity 4**<br>6.00 - 6.25<br>**(25 minutes)**	**Whole group discussion**<br>Facilitator guides discussion using questions below as prompts. These can be added to chat if needed – *20 minutes*<br>1. If a friend of your age and gender was going to the doctor for the first time, what would you tell them to expect? (10 minutes)<br>2. What should a consultation with a healthcare professional be like, in the most ideal situation? (10 minutes)<br><br>*Final question done as a chat waterfall*<br>3. If you could tell healthcare professionals one positive change they could make to improve young women’s healthcare experiences, what would it be? (chat waterfall – 5 minutes)	**Facilitator 3 facilitates with other facilitators adding to discussion and supporting young people to engage.**<br><br>**Facilitator 2 chat waterfall.**	**Check we are recording for group discussion**<br><br>**Slides 20-23 if wanting to use them. Otherwise, Facilitator 1 to put them in the chat**<br><br>**Facilitator 1 to manage time to allow discussion of each question**<br><br>Chat waterfall for the final question
**Cool down & close**	In the chat<br>- A word, phrase or emoji to describe your experience	**Facilitator 1**	Using the chat

6.20 - 6.30<br><br>**(10 minutes)**	today<br>- Second feelings check in<br>**Facilitator 1 to:**<br>- Outline next steps in the study and post link for the feedback form<br>- Let us know in the chat if you are interested in the training<br>- Thank contributors for their time and engagement. Share the resources<br>- Post session all facilitators to debrief and identify any concerns		
