# Supplementary material for: Co‐Producing Training Resources to Improve Healthcare Experiences for Young Women and Reduce Gendered Health Inequalities
Source: Health Expect. 2026 Aug 20;29(4):e70834. doi: 10.1111/hex.70834 (PMC13492181; doi:10.1111/hex.70834)
Supplement: Supplementary file 3 [file HEX-29-e70834-s003.pdf]

Improving Young Women's Care

Resources for healthcare professionals

SHIFT

Improving healthcare experiences for young women

Understanding Young Women's Health Experiences

Our research found that many young women felt their health concerns were dismissed or not believed by healthcare professionals. Stereotypes of young women as naïve, overdramatic, hormonal, irrational and stupid were regularly experienced, leading them to feel patronised, not listened to and not taken seriously. The project enabled young women to discuss the key things that were important for a positive healthcare experience.

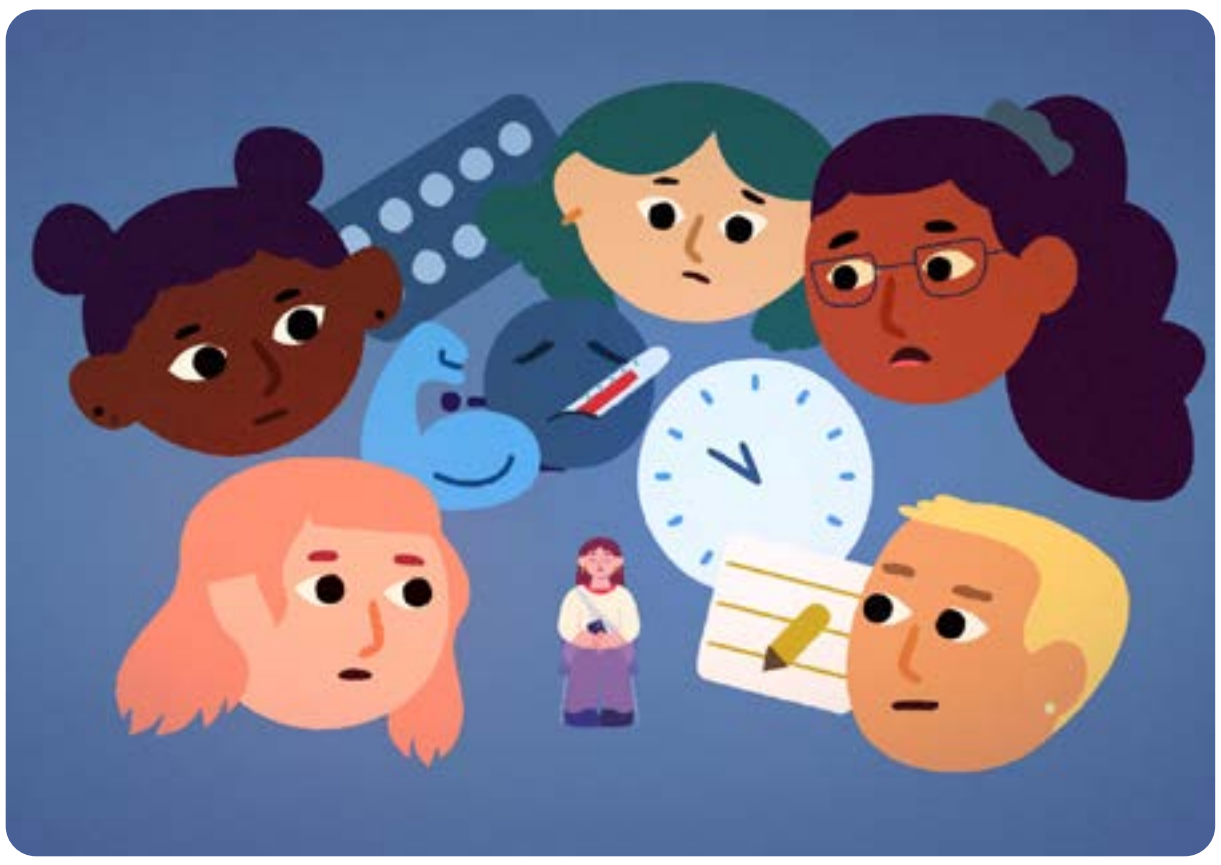

About the Work

Together with young women and healthcare professionals we co-produced an animation and training workshop to improve healthcare experiences for young women. The project team included researchers from the University of Bristol, the Women's Health Plan Scotland Team at the Scottish Government, The Young Women's Movement and the Association for Young People's Health.

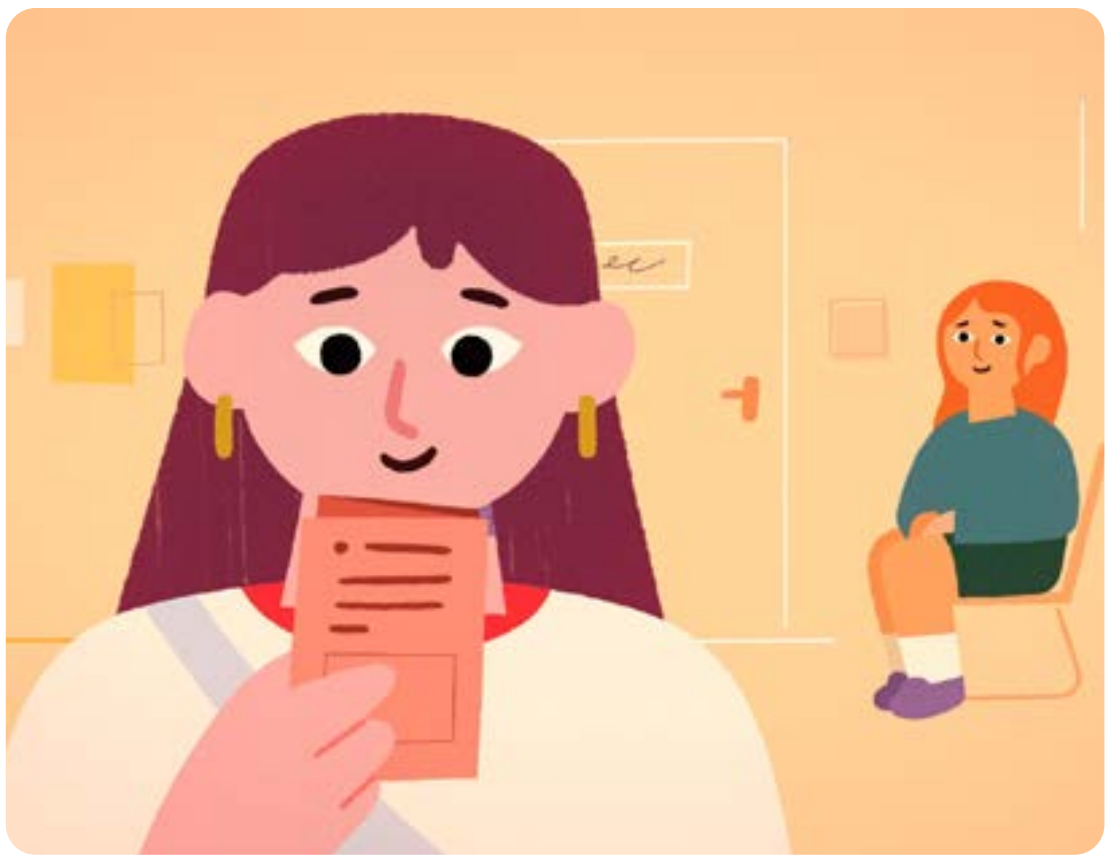

Access the Resources

View the animation here or scan the QR code!

It is 2.5 minutes long. Please share it far and wide!

We also trained a group of young women to co-facilitate training sessions for healthcare professionals. **Contact us** if you would like to access training workshops for your team or area.

Find Out More

- If you have questions about the research, contact: **laura.tinner@bristol.ac.uk**
- To book a training session co-facilitated by young women, contact: **info@ayph.org.uk**

Funded by:

UKRI Economic and Social Research Council

NIHR | School for Public Health Research

In partnership with:

The Young Women's Movement

Scottish Government Riaghaltas na h-Alba

ayph

media co-op

University of BRISTOL
