# Supplementary material for: Co‐Producing Training Resources to Improve Healthcare Experiences for Young Women and Reduce Gendered Health Inequalities
Source: Health Expect. 2026 Aug 20;29(4):e70834. doi: 10.1111/hex.70834 (PMC13492181; doi:10.1111/hex.70834)
Supplement: Supplementary file 4 [file HEX-29-e70834-s004.pdf]

View in browser

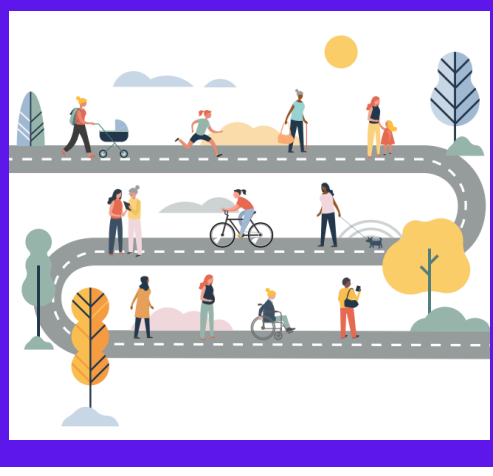

Steering Committee Update

Dear {{ contact.FIRSTNAME }},

Hope you're doing well and enjoying the warmer weather. We wanted to share a quick update as lots has happened!

We've now completed the workshops with young women and healthcare professionals (HCPs), and we've started working with [redacted] on developing the resource. After great input from the workshop with healthcare professionals, we've decided to create an **animation** as the final format.

Jenni from media co-op, who's leading this part of the project, has drafted a first version of the **script (attached)**. We'd really value your thoughts, so please take a moment to review it and share any feedback using this **short form**. If you could send feedback by **Tuesday 18th July** that would be really helpful.

Read on below for more info about the workshops, animation brief, and our upcoming meeting on the 24th July, 11-12.

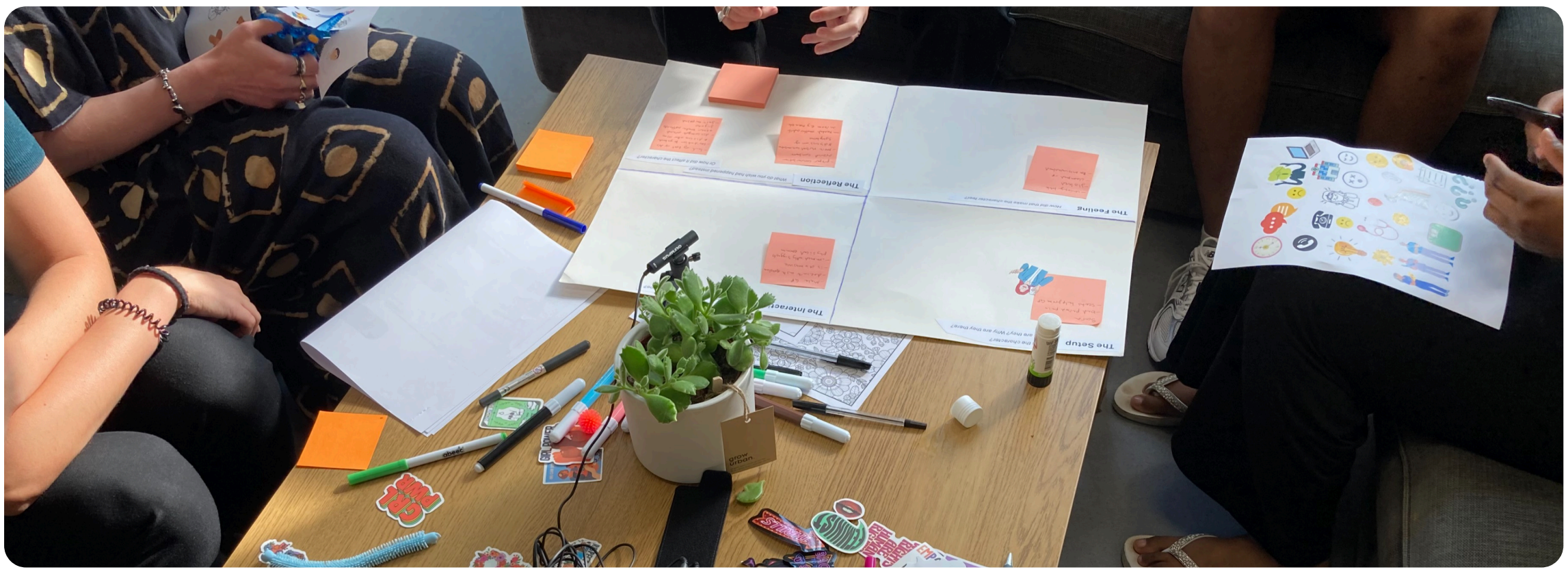

Young Women's Workshop (24 participants)

Young Women's - Online: 9
In-person (The Junction): 10
In-person (GWL): 5

Across all workshops with young women, three key themes emerged from their reflections on healthcare experiences:

- **Feeling heard & respected:** Young women often felt dismissed, which stopped them from speaking openly. Starting from a place of belief and respect, even without clear answers, made a big difference.
- **Choice & clarity:** Young women want options and value being included in decisions, clear explanations and healthcare that looks at their whole lives, not

just reproductive health.
- **Ongoing care & advocacy:** Young women want to feel supported over time, not just in one appointment. Positive experiences involved follow-up, open dialogue and support to advocate for themselves, especially as many are navigating healthcare independently for the first time.

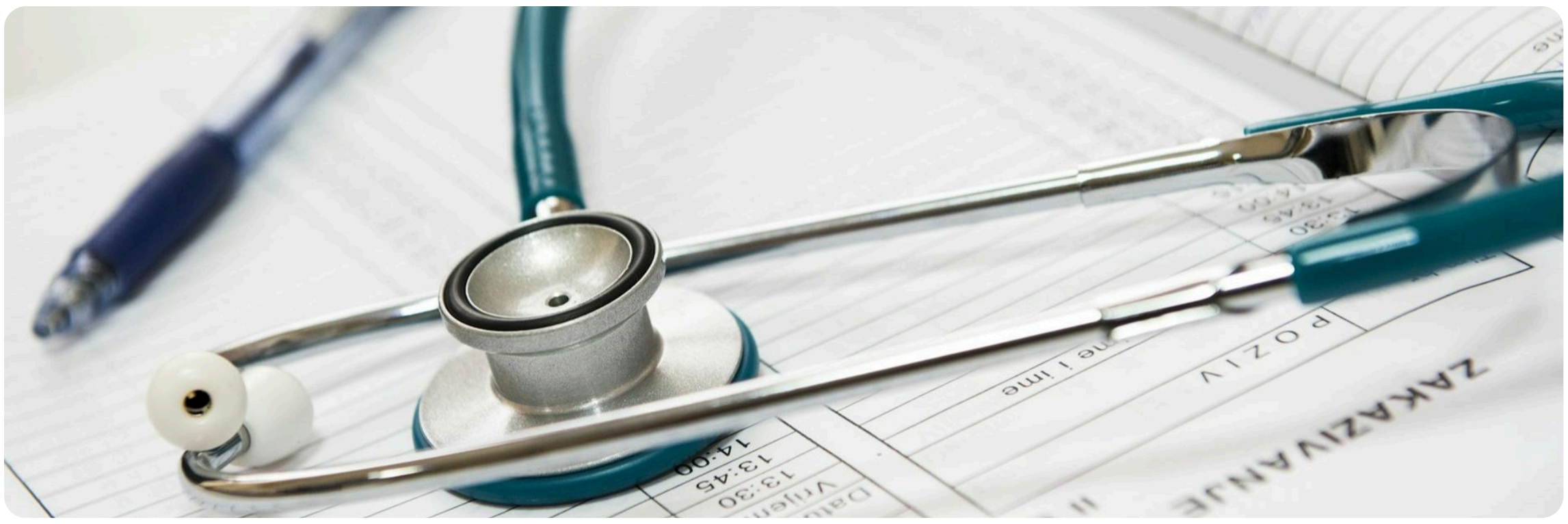

Healthcare professional's workshop (13 participants)

Healthcare professionals - Online: 13

We shared these key themes from the young women's workshops with a group of HCPs. The discussion focused on how to best translate these insights into a practical and meaningful resource.

There was strong agreement that the resource should:

- **Be short and to the point**, recognising the time pressures healthcare professionals face. It should quickly convey the most important messages without being overwhelming.
- **Be accessible for both HCPs and young people**, with language and visuals that are clear, inclusive and easy to engage with.
- **Have a light-hearted tone**, where appropriate, to help make the topic approachable and avoid making the resource feel too heavy or formal.
- **Be non-judgemental**, acknowledging the systemic challenges and pressures within healthcare. The aim is not to criticise individual staff but to offer helpful suggestions and perspectives that support better interactions with young patients.

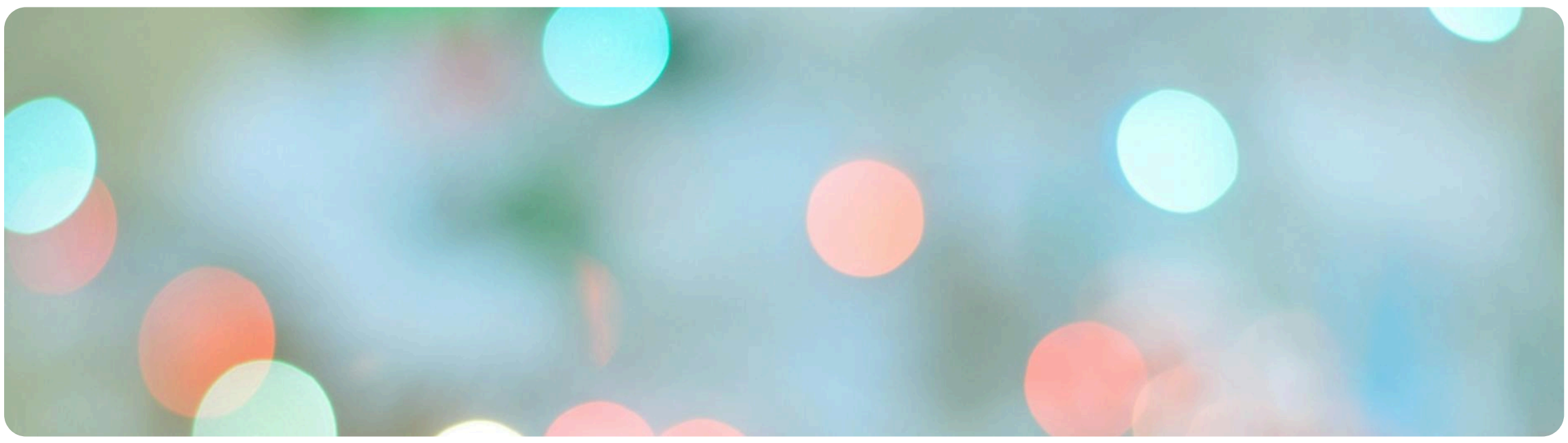

Animation

Following feedback from healthcare professionals, [redacted] who specialise in creating engaging, accessible resources, recommended producing an animation.

They proposed the following brief:

- A **2-minute animation** capturing young women's real experiences of healthcare
- Focused on how healthcare interactions **make young women feel**

- Highlighting both **positive and negative behaviours** from healthcare professionals
- **Aimed at healthcare professionals.**

[redacted] have now developed a draft script (attached) for the animation and would really value your feedback at this stage. Once the animation moves into production, it will be much harder to make changes, so this is a key moment to shape the final resource.

Script: Feedback Form

FEEDBACK DEADLINE: next Tuesday 15th!

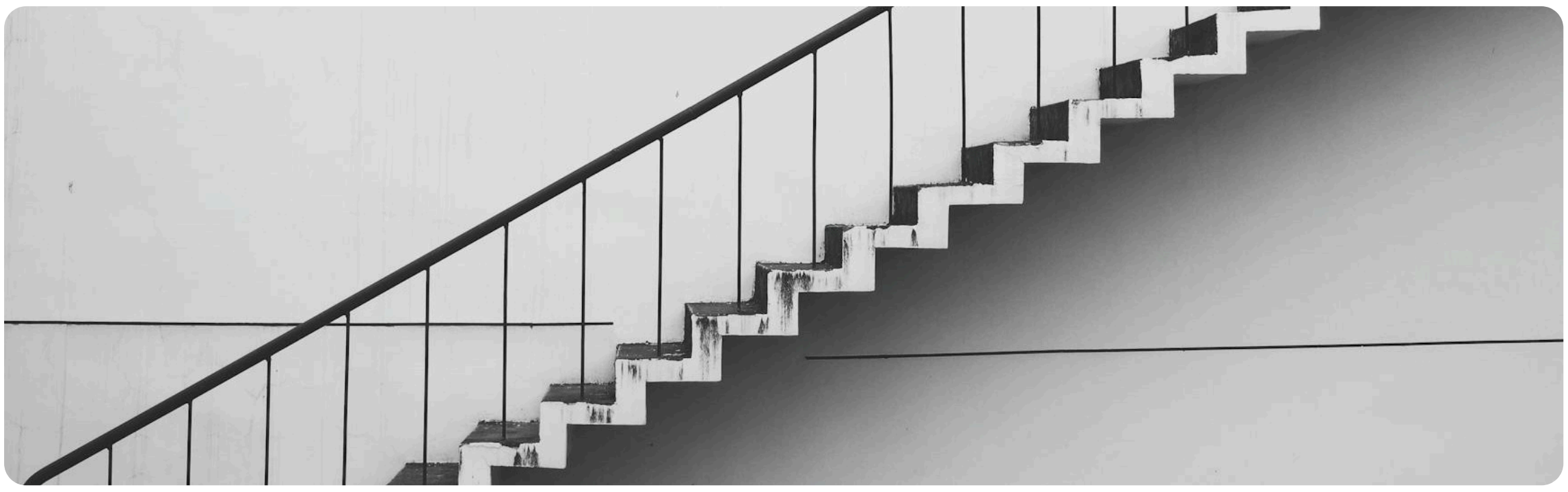

Next Meeting

Our next meeting is scheduled for **Thursday 24th July at 11am-12pm**—please save the date! You should have received a calendar invite by now, let us know if you haven't. We will send out an email closer to the time with the agenda.

Just a heads up [redacted] will be joining us at the meeting too, to hear your thoughts on some of the visuals they are creating.

Link to meeting

24th July
11am -12pm

Thank you again for all your input and engagement. If anything else comes to mind, just drop us a message, excited to keep this work moving forward together.

Best,

The Team

This project is funded by the Economic and Social Research Council and NIHR School for Public Health Research
