# Supplementary material for: Co‐Producing Training Resources to Improve Healthcare Experiences for Young Women and Reduce Gendered Health Inequalities
Source: Health Expect. 2026 Aug 20;29(4):e70834. doi: 10.1111/hex.70834 (PMC13492181; doi:10.1111/hex.70834)
Supplement: Supplementary file 5 [file HEX-29-e70834-s005.pdf]

View in browser

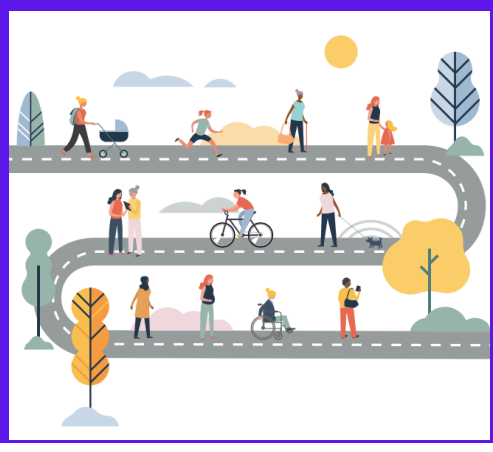

Steering Committee Meeting Recap (5th March)

Dear {{ contact.FIRSTNAME }},

Thanks for attending our first committee meeting, and for making the time to fill out the brief survey beforehand. It was great to see so many responses there and to hear all your thoughts and feedback at the meeting. We're very happy that you are part of this project!

This is just a short email to recap what we discussed in the meeting. Feel free to get in touch if you'd like to add anything or follow up on any thoughts.

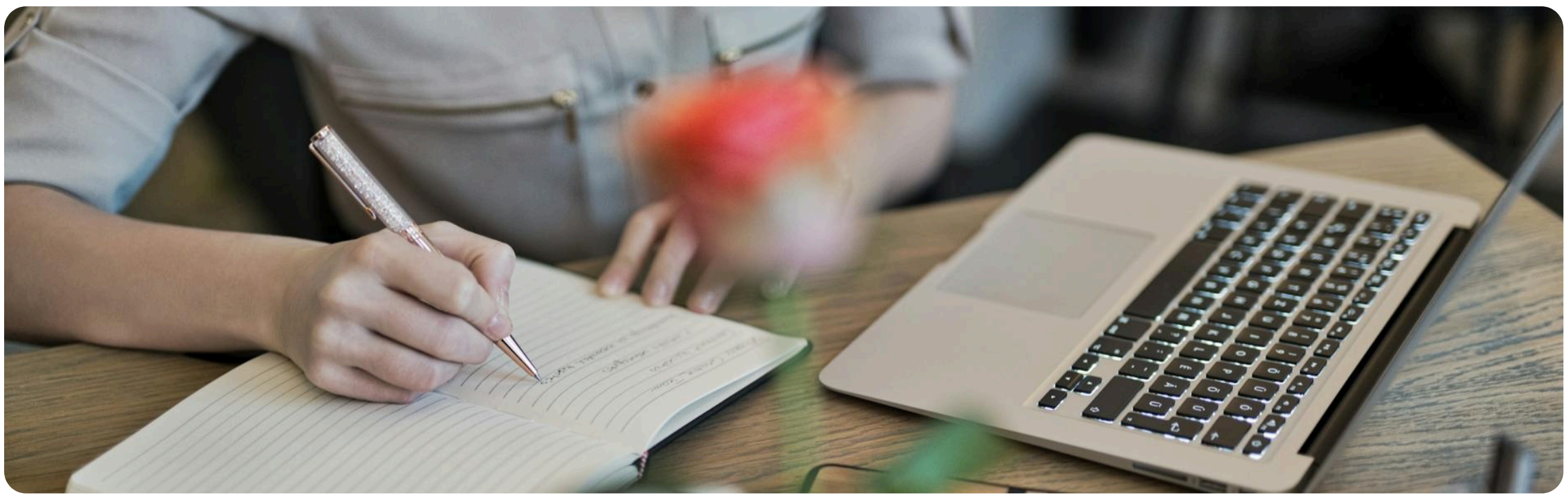

Meeting Recap

Below is a quick recap of what we discussed:

- **Project Overview and Terms of Reference**[redacted] provided an overview of the project and shared some of the papers with the research. We collectively agreed on the terms of reference (attached in our last email!), which describes what your role is and how we will all work together.

- **Young Women's Workshops:**
    - **Recruitment Strategy:** Based on survey responses and discussion, we agreed there are lots of groups of young women who are particularly discriminated against by healthcare professionals or marginalised by healthcare. Trying to include them all is a task and we don't want to over promise. We will work with community groups to target recruitment (instead of an open call), keeping deprivation, ethnicity, LGBTQ+ and disability at the fore of our strategy. Working with a specific group facing multiple disadvantage (e.g., care experienced young women, young mothers) is one strategy for capturing that intersectional perspective. We will also use the online session to ensure inclusion of rural young women and those with access needs.
    - **Key Considerations:** Ensuring accessibility is key—this includes addressing language and cultural sensitivities and childcare expenses. Working closely with trusted community groups within places of deprivation will go some way to

reaching our target populations. We will be transparent about the project aims and confidentiality.

- **Healthcare Professional Workshops:**
  - **Recruitment Strategy:** Emphasis was placed on community health workers and clinical support workers, particularly in primary care settings. Youth organizations and platforms like NHS Inform, Practice Managers, and local community centres (e.g., Citizens Advice Bureau) were suggested as key venues for disseminating information and recruiting participants. Some of our steering committee members also have good networks for recruitment. It was suggested to include young HCPs, especially those in training, as they might be more open to change and be able to disseminate if they are involved in teaching.
  - **Key Considerations:** The time and capacity pressures faced by healthcare professionals need to be considered, with meetings requiring adequate notice (at least 6 weeks) and practice backfill for staff coverage. Avoiding summer holidays will be useful. We want to make efforts recruit HCPs who are least likely to change.

- **Project Name:** *Her Health Matters* and *SHIFT* got the most votes, but concerns were raised about inclusivity and reservations about the use of "Her" in the name, as it could feel exclusionary to some groups. We're taking all your feedback into account before making a final decision.

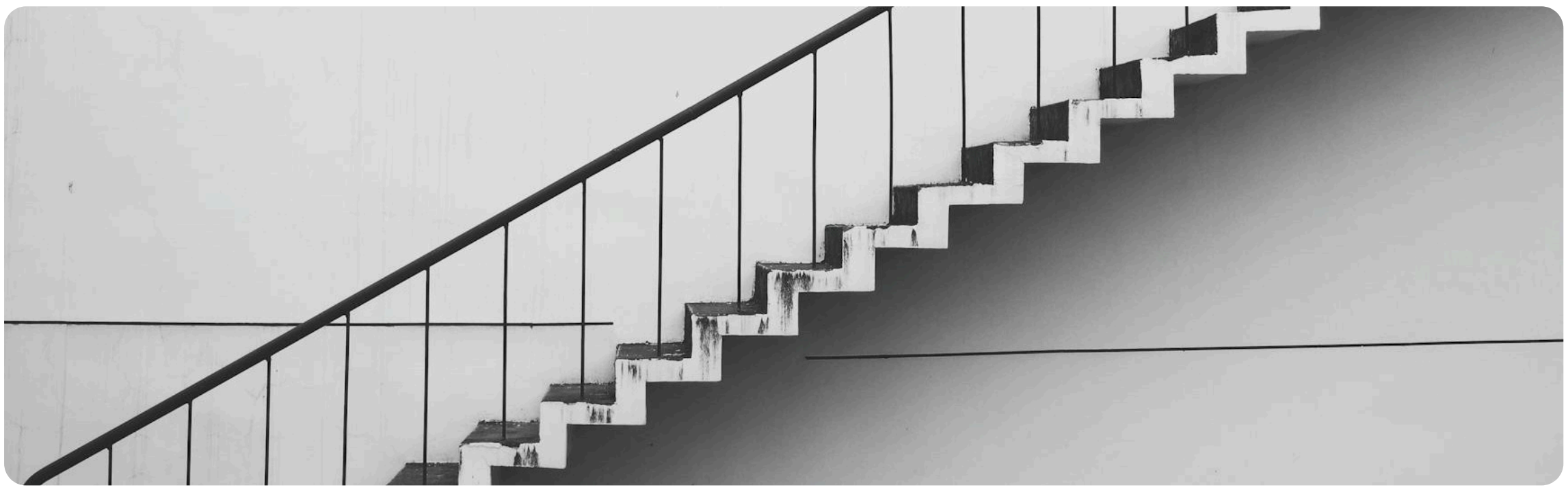

Next Meeting

Our next meeting is scheduled for **Wednesday April 30th (11 AM - 12 PM)**—please save the date! You should have received a calendar invite by now, let us know if you haven't. We will send out an email closer to the time with the agenda, short pre-meeting survey and meeting link.

Link to meeting

30th April
11am -12pm

Thank you again for all your input and engagement. If anything else comes to mind, just drop us a message—excited to keep this work moving forward together.

And if you have any feedback on our first meeting, feel free to let us know by responding to this or emailin[redacted]—we want to make

these as useful as possible.

Best,

The Team

This project is funded by the Economic and Social Research Council and NIHR School for Public Health Research
