# Supplementary material for: Co‐Producing Training Resources to Improve Healthcare Experiences for Young Women and Reduce Gendered Health Inequalities
Source: Health Expect. 2026 Aug 20;29(4):e70834. doi: 10.1111/hex.70834 (PMC13492181; doi:10.1111/hex.70834)
Supplement: Supplementary file 6 [file HEX-29-e70834-s006.pdf]

Preview mode

Published

Copy responder link

Steering Committee meeting - 5th March 2025 - 4.30pm

Hi there! Thanks for being part of this project, we're looking forward to meeting you all.

We've created this brief survey to ensure we make the most of our meeting next week. We won't single anybody out or tell the others what you said, this is purely to get the conversation started and to make sure everyone has been able to have a say. If you can't think of anything for a question, you can leave it blank.

We would also like to see when everyone is free for the next meeting.

What is the purpose of the project?

- This project aims to address discrimination in healthcare experienced by young women* in Scotland. Research in 2023 found that many young women felt their health concerns were dismissed due to harmful stereotypes.
- To tackle this, we are co-creating training resources—such as animations and podcasts—with young women (aged 16-25) and healthcare professionals in Scotland
- There will be 3 workshops with young women and 1 with healthcare professionals (a mixture of online and in person)

(*Inclusive of trans and intersex women, as well as non-binary and gender-fluid people comfortable in a space centring women's experiences)

* Indicates required question

Preview mode

Published

Copy responder link

Your answer

Role or interest in the project
(E.g., Nurse, Lived Experience Member, GP).

This will appear on the list of Steering Committee members in the Terms of Reference only, so that everyone can see where everybody is coming from. This will not be used to group people and we will not be focusing on these roles within the meeting. Everyone's contribution is equal.

Your answer

Which groups of young women would benefit the most from being included in this project?

Are there specific communities or experiences we should prioritise—such as young women with disabilities, care-experienced young women, LGBTQ+ young women, or those from minority ethnic backgrounds? We'd love to hear your thoughts on making this as inclusive and impactful as possible!

Your answer

Do you know of any organisations, networks, or community groups that could help us engage young women (16-25) in Scotland—either to connect with participants or as potential workshop hosts?

This could include youth organisations, advocacy groups, student networks, or community centres. Any recommendations would be really helpful!

Your answer

Preview mode Published Copy responder link

These may be groups you feel would benefit from training on young women friendly services, or professionals that young women come into contact with a lot. By healthcare professionals, we mean GPs, Practice Nurses, Sexual Health professionals, Mental Health professionals etc.

Your answer

At our steering committee meeting, we'll check your availability for our next meeting. Below are some options—**please select all the times that work for you!**

- [ ] Tuesday 29th April, 10-11
- [ ] Tuesday 29th April, 11-12
- [ ] Tuesday 29th April, 14-15
- [ ] Tuesday 29th April, 16:30-17:30
- [ ] Tuesday 29th April, 17:30-18:30
- [ ] Wednesday 30th April, 10-11
- [ ] Wednesday 30th April, 11-12
- [ ] Wednesday 30th April, 14-15
- [ ] Wednesday 30th April, 16:30-17:30
- [ ] Wednesday 30th April, 17:30-18:30

You can find the meeting agenda here.

Feel free to let us know if you have any thoughts on things related to this project that we should discuss in future, no worries if not!

Your answer

Preview mode

Published

Copy responder link

This content is neither created nor endorsed by Google. - Contact form owner - Terms of Service - Privacy Policy

Does this form look suspicious? Report

Google Forms

Preview mode

Published

Copy responder link
