# Supplementary material for: Co‐Producing Training Resources to Improve Healthcare Experiences for Young Women and Reduce Gendered Health Inequalities
Source: Health Expect. 2026 Aug 20;29(4):e70834. doi: 10.1111/hex.70834 (PMC13492181; doi:10.1111/hex.70834)
Supplement: Supplementary file 7 [file HEX-29-e70834-s007.docx]

**Improving the quality of healthcare for young women**

**Steering Committee Terms of Reference**

Project: Co-producing resources for healthcare professionals to reduce discrimination and improve quality of healthcare for young women

Funders: [Economic and Social Research Council](https://www.ukri.org/councils/esrc/) and [NIHR School for Public Health Research](https://sphr.nihr.ac.uk/)

*Goals and scope*

The Steering Committee for this project will serve the following functions:

1. To attend 5 meetings, that will be structured around a topic, providing advice and support, occasionally commenting on documents outside of meetings.
2. To act as a ‘critical friend’, giving feedback and being involved in discussion to help make the project successful.
3. To assist the project team to identify opportunities for further collaboration and funding.
4. To raise the profile of the project, assist with dissemination of outputs and act as ambassadors and advocates for the project.

The contributions of the Steering Committee will vary and evolve as the project progresses, but may involve:

- Advice on groups to engage in the workshops, suggestions for population groups to prioritise (of both young women and healthcare professionals).
- Supporting the development of the co-produced resource – e.g., the Steering Committee may have content and style ideas, ideas on format and where we might show it.
- Highlighting other projects, events and policies ongoing where we could make meaningful contributions or collaborate.
- Suggesting different avenues for dissemination and impact.

*Membership and appointment*

The group will consist of approximately 8 members who will serve for the duration of the project. Members will be appointed through team contacts, aiming for diversity of perspective on young women’s health. Members will represent practice, policy, research, direct experience, and voluntary and community organisations.

Members will endeavour to take part in all meetings and where appropriate, send a deputy if they are unable to attend. Members may invite other people to attend meetings if they feel their perspective will be valuable, but this should be discussed with the group.

The Project Team will also attend the Steering Committee meetings where possible and appropriate.

*Meeting structure*

Each meeting will consist of an agenda, sent by the project team ahead of the meeting, with timings that will be led by the Chair. We have a broad plan for the focus of meetings, which will be circulated at the first meeting.

*Meeting expectations*

As a group we will all agree to the following to ensure safe, respectful and productive meetings:

- Everyone's voice is valid and everyone's contribution equal.
- Whilst it is not a requirement it is possible that we may share lived experiences, so being respectful of each other and looking after ourselves is important. This may include taking a time out during the meeting, turning cameras off or asking for further support from one of the project team.
- Nobody has to share anything they don’t want to and anything you do share, the rest of the group will respect and not repeat outside of the meeting unless they have permission to do so.
- Stay on mute if not speaking to avoid background noise.
- We have a limited time together, so the Chair will keep people to time and may move people on. You can contact us via email after meetings if you feel you have more to share.
- Please feedback if something in the meeting isn't working for you. We are all learning in the process together and remain adaptable based on what works best for the group.

*Chairing, frequency and reporting*

Meetings will be chaired by one of the Project Team, with the option to circulate the Chair as the Committee prefers.

Over the 9-month project (running January – September 2025) there will be approximately 5 meetings, adjusted based on need and capacity of the members.

Each meeting will last for 1 hour and will be online, unless we are able to arrange an in-person meeting that everyone can attend.

There may be some preparation or reflection work to be undertaken outside of meetings, but this will be highlighted in advance and will be kept to a minimum.

One of the project team will keep confidential (and brief) minutes and circulate these to the group after the meeting.

*Payment and expenses*

There is funding to reimburse all members for their time at the rate of £25 per hour (the NIHR Public Involvement rate), plus any travel expenses should we meet in person. We appreciate that some job roles will not be able to take up this offer. A University of Bristol payment form will be circulated before the first meeting to collect the bank details for payments. These will be stored securely at the University of Bristol. Laura will confirm with everyone that there have been no changes to the details and process the payment forms promptly after each meeting.

*Project team*

This is the team who proposed the project and are working together to make it a success. We’ll be creating the documents, running the Steering Committee meetings and facilitating the workshops. If you are unsure of something, feel free to ask one of us. We are here to support you as a committee.

*Table removed for anonymisation for publication.*

*Steering Committee Members*

These are the Steering Committee members.

Table removed for anonymisation for publication.
